# Multimodal Registration for Image-Guided EBUS Bronchoscopy

**DOI:** 10.3390/jimaging8070189

**Published:** 2022-07-08

**Authors:** Xiaonan Zang, Wennan Zhao, Jennifer Toth, Rebecca Bascom, William Higgins

**Affiliations:** 1 School of Electrical Engineering and Computer Science, Penn State University, State College, PA 16802, USA; xiaonanzang@gmail.com (X.Z.); wfz5024@psu.edu (W.Z.); 2 Penn State Milton S. Hershey Medical Center, Hershey, PA 17033, USA; jtoth@pennstatehealth.psu.edu (J.T.); rbascom@pennstatehealth.psu.edu (R.B.)

**Keywords:** bronchoscopy, endobronchial ultrasound, image registration, image-guided surgery systems, multimodal imaging, lung cancer

## Abstract

The state-of-the-art procedure for examining the lymph nodes in a lung cancer patient involves using an endobronchial ultrasound (EBUS) bronchoscope. The EBUS bronchoscope integrates two modalities into one device: (1) videobronchoscopy, which gives video images of the airway walls; and (2) convex-probe EBUS, which gives 2D fan-shaped views of extraluminal structures situated outside the airways. During the procedure, the physician first employs videobronchoscopy to navigate the device through the airways. Next, upon reaching a given node’s approximate vicinity, the physician probes the airway walls using EBUS to localize the node. Due to the fact that lymph nodes lie beyond the airways, EBUS is essential for confirming a node’s location. Unfortunately, it is well-documented that EBUS is difficult to use. In addition, while new image-guided bronchoscopy systems provide effective guidance for videobronchoscopic navigation, they offer no assistance for guiding EBUS localization. We propose a method for registering a patient’s chest CT scan to live surgical EBUS views, thereby facilitating accurate image-guided EBUS bronchoscopy. The method entails an optimization process that registers CT-based virtual EBUS views to live EBUS probe views. Results using lung cancer patient data show that the method correctly registered 28/28 (100%) lymph nodes scanned by EBUS, with a mean registration time of 3.4 s. In addition, the mean position and direction errors of registered sites were 2.2 mm and 11.8${}^{\circ }$, respectively. In addition, sensitivity studies show the method’s robustness to parameter variations. Lastly, we demonstrate the method’s use in an image-guided system designed for guiding both phases of EBUS bronchoscopy.

## 1. Introduction

**Figure 1:**
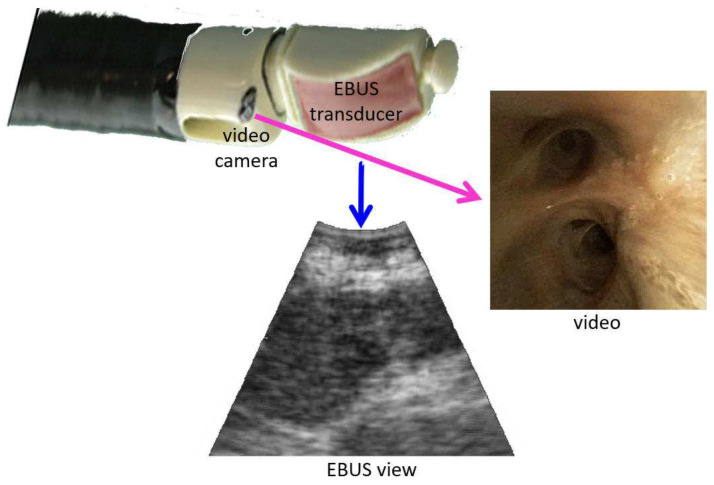
Device tip and example views for an integrated EBUS bronchoscope. The video camera provides bronchoscopic video, while the EBUS transducer gives 2D EBUS views. The magenta and blue arrows correspond to videobronchoscope camera axis $n_{C}$ and EBUS probe axis $n_{\mathrm{US}}$, respectively.

The state-of-the-art procedure for examining the lymph nodes in lung-cancer patients draws on an endobronchial ultrasound (EBUS) bronchoscope [1,2,3,4]. The EBUS bronchoscope, also referred to as a convex-probe EBUS or linear EBUS bronchoscope, integrates two modalities into one device (Figure 1): (1) videobronchoscopy, which gives video images of the airway walls (endoluminal surfaces); and (2) convex-probe EBUS, which gives 2D fan-shaped views of extraluminal structures situated outside the airways. Due to the fact that lymph nodes lie outside the airways—hence, are unobservable by videobronchoscopy—EBUS is essential for confirming a node’s location.

**Figure 2:**
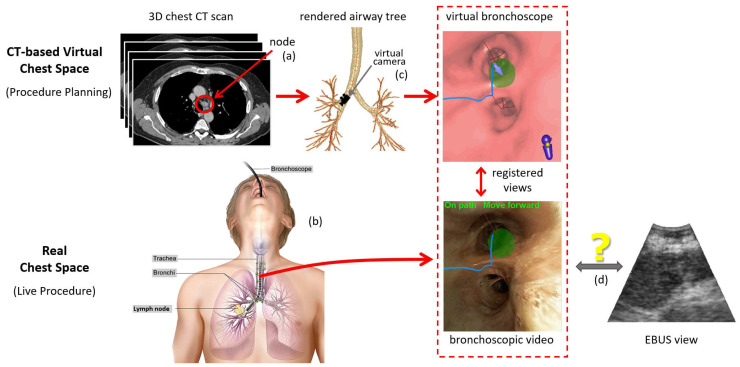
Overview of an image-guided EBUS bronchoscopy procedure. Top part of the figure focuses on the 3D CT-based virtual chest space, whereas the bottom part features the analogous real 3D chest space. (**a**) Before the live procedure, the physician selects lymph nodes of interest from a patient’s chest CT scan. (**b**) During the live surgical procedure, the physician then uses this plan to navigate the bronchoscope toward the lymph node. (**c**) When using an image-guided bronchoscopy system, the physician receives additional graphical feedback on how to navigate the device toward the lymph node. VB views situated along a precomputed guidance path (blue line) lead the physician toward the lymph node. Image registration between the CT-based VB views and live bronchoscopic video (red dotted-line box) facilitates device synchronization during navigation and leads the physician to the proper airway closest to the node (green region). (**d**) Existing image-guided bronchoscopy systems offer no means for helping to place the EBUS probe for live localization of the extraluminal lymph node. (Bronchoscopy drawing by Terese Winslow, “Bronchoscopy,” NCI Visuals Online, National Cancer Institute.)

Before the procedure, the physician first examines a patient’s 3D chest computed tomography (CT) scan to select suspicious lymph nodes (Figure 2a). Later, during the live surgical procedure, the physician uses the EBUS bronchoscope to localize and biopsy the lymph nodes (Figure 2b). In particular, for each node, the physician first employs videobronchoscopy to navigate the device through the airways. Next, upon reaching the node’s approximate expected vicinity, the physician probes the nearby airway walls using EBUS to localize the node.

Unfortunately, EBUS can be difficult to use effectively [5]. This situation arises for three reasons [6,7,8]. First, the physician must mentally translate their anatomical knowledge to CT scan observations and live EBUS views. Second, EBUS views generally do not lie on the orthogonal 2D axial, coronal, or sagittal planes readily observed in CT. This makes it hard to correlate CT observations to live EBUS views. Finally, because of EBUS’s limited field of view (FOV), the physician performs an essentially blind trial-and-error EBUS sweep to localize a node. This can be challenging given the typical small size of lymph nodes (≈10 mm long-axis length) and the complex 360${}^{\circ }$ cylindrical span of the airway walls.

Recently proposed image-guided bronchoscopy systems have proven to make videobronchoscopic navigation through the airways a high-success, skill-independent operation [9,10]. Using the concept of virtual bronchoscopy (VB), the live bronchoscopic video can be readily correlated and registered to precomputed CT-based VB views that mimic the video views of the real bronchoscope’s video camera [11,12,13,14]. Figure 2c illustrates this operation during a live procedure. The physician follows the guidance system’s presented sequence of VB views along a preplanned guidance route, which, in turn, leads the physician to each diagnostic site. Along the way, image registration synchronizes the position of the real device and virtual camera.

These systems, however, do not offer any means to guide the placement of the EBUS probe for localizing extraluminal sites, as suggested by Figure 2d. Thus, accurate localization, crucial for effective biopsy, continues to be problematic [8,15]. To emphasize this point, a multi-physician study showed that a physician’s biopsy success rate was only 43% despite successfully navigating to the correct airway 78% of the time [16]. Other studies have shown that, even when image-guided navigation and EBUS are used together, biopsy success rates barely >50% still occur [17,18]. We propose a method for registering the pre-operative chest CT scan to the live EBUS views, thereby facilitating accurate image-guided EBUS bronchoscopy.

Several recent works have attempted to extend image guidance to EBUS localization [19,20,21,22,23]. For EBUS bronchoscopy as applied to lymph node examination, the approach of Sato et al. entails considerable manual interaction to preplan sites for invoking EBUS, and offers no virtual-to-real EBUS registration to help with EBUS localization [19]. Sorger et al. proposed a commendable electromagnetic navigation system that draws upon an extra sensor attached to the EBUS bronchoscope [20,21]. However, the system does not register the live EBUS probe view to the pre-operative CT, thereby limiting optimal EBUS placement. It also adds the well-known issues arising from using electromagnetic technology, namely the extra device cost and susceptibility to patient motion [24].

The other works applied virtual bronchoscopy to the allied problem of peripheral nodule diagnosis [22,23]. The physician uses VB to navigate a standard videobronchoscope close to the nodule and then inserts a radial-probe EBUS, which gives 360${}^{\circ }$ views of the extraluminal airway structure, into the videobronchoscope’s working channel. Since radial-probe EBUS is not designed for examining central-chest lymph nodes, it presents a different guidance scenario (separate devices, requires probe removal before biopsy, dissimilar imaging scenario) [1]. This research, however, does give impetus for work with convex-probe EBUS. The approach of Tamiya et al. does not give direct guidance for EBUS placement [22]. The method of Luo and Mori does accomplish EBUS localization. However, it needs extra sensors to track the device and was only tested in a controlled phantom environment [23].

Our proposed registration method draws upon the concept of a CT-based virtual EBUS, as introduced by Zang et al. [25,26]. The virtual EBUS, which mimics the FOV of the convex-probe EBUS, acts as a complementary analog to the virtual bronchoscope used for image-guided navigation. With the virtual EBUS and associated registration method, live image-guided EBUS probe localization can now be performed.

**Figure 3:**
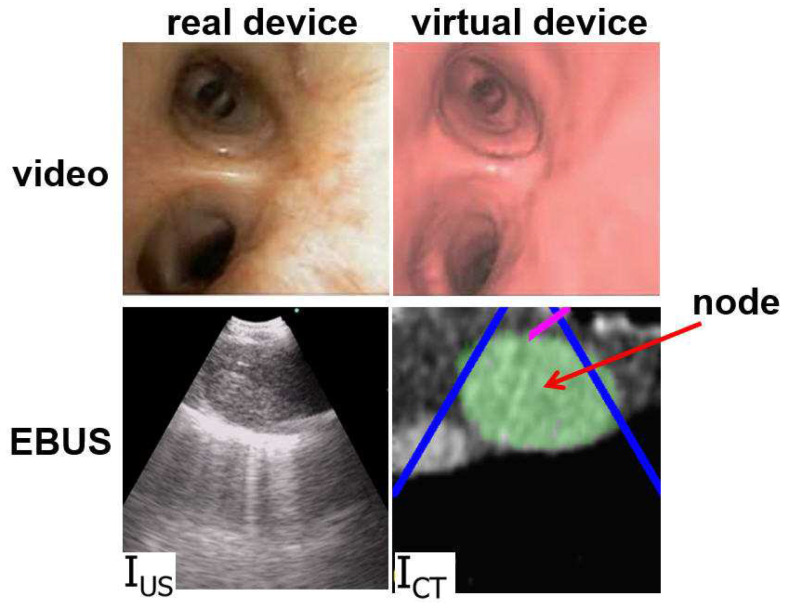
Example videobronchoscope and EBUS views constituting the multimodal EBUS bronchoscope. (**Top**) bronchoscopic video sources for the real EBUS bronchoscope and virtual device. (**Bottom**) corresponding fan-shaped EBUS views, $I_{\mathrm{US}}$ and $I_{\mathrm{CT}}$, for the real and virtual devices, respectively. For virtual EBUS view $I_{\mathrm{CT}}$, the blue lines demarcate the EBUS FOV, the magenta line indicates the video camera’s viewing direction $n_{C}$, and the green region denotes a lymph node predefined in the chest CT scan. In this figure, the videobronchoscope and EBUS view pairs are not at the same site. In addition, both view pairs are registered.

The method, partially motivated by previous research in ultrasound registration, draws upon the idea of mutual information (MI). MI techniques have been commonly used to register ultrasound and CT images since the MI metric does not require the two sources to have similar image appearances [27,28]. Figure 3, discussed in Section 2, clearly illustrates how the real and virtual EBUS views differ in appearance. Unfortunately, because the MI metric relies on a joint intensity probability model, while completely ignoring spatial information, MI-based techniques can fail when shading artifacts exist [29,30]. To improve MI-based methods, Studholme et al. proposed a normalized MI (NMI) metric, which is more robust to image overlap [31]. They also integrated spatial information by calculating mutual information among local regions [32]. As a further enhancement, researchers have included prior knowledge, such as the region shape, to achieve more robust accurate registration. For example, Knops et al. performed k-means clustering to distinguish image pixels having similar intensities but belonging to different regions. They then used these modified intensity bins to calculate NMI during registration [33].

During live EBUS localization, our proposed method uses an optimization process, which combines both NMI and region shape information, to register CT-based virtual EBUS views to the live views generated by the real EBUS probe. We also integrated the method into an image-guided EBUS bronchoscopy system that features a multimodal virtual EBUS bronchoscope, which integrates both the standard virtual videobronchoscope and the virtual EBUS [26]. Thus, intuitive image guidance is now attainable for both videobronchoscopic navigation and EBUS localization. Section 2 details the method. Section 3 validates the method performance using lung cancer patient data. It also illustrates the method’s utility in our image-guided EBUS bronchoscopy system. Finally, Section 4 offers concluding comments.

## 2. Methods

### 2.1. Overview

We first overview the protocol employed by our image-guided EBUS bronchoscopy system [26]. Section 2.2 then discusses the method for registering the virtual EBUS to live EBUS probe views.

To begin, the system complied with the standard protocol of nodal staging bronchoscopy. First, prior to the procedure, a guidance plan was derived from the patient’s high-resolution 3D chest CT scan (voxel dimensions Δx,Δy,Δz< 1 mm) using methods similar to those employed by existing image-guided bronchoscopy systems [9,24,34]. This gives the airway tree’s endoluminal surfaces, a set of target lymph nodes, and an airway guidance route for each node. We used previously validated methods to generate the guidance plan [25,34,35,36,37].

Next, in the operating room, the EBUS bronchoscope was interfaced to the guidance computer. It was assumed that the physician uses the Olympus BF-UC180F EBUS bronchoscope, the de facto standard device for EBUS nodal staging [1,3]. This device produces data streams for both the videobronchoscope and EBUS probe (Figure 1), which serve as system inputs. Section 2.3 gives technical detail for the guidance computer and interfacing to the bronchoscopy suite.

Image-guided EBUS bronchoscopy fundamentally involves aligning planning data derived from the patient’s chest CT scan to live views provided by the EBUS bronchoscope. This requires the definition of virtual and real spaces representing the chest (Figure 2). For our scenario, the 3D chest CT scan defines a virtual chest space, whereby a virtual EBUS bronchoscope moves through the CT-based virtual airways. Similarly, as the real device navigates through the physical airways, the EBUS bronchoscope’s data streams give views inside the corresponding real 3D chest space. This is similar to what current image-guided bronchoscopy systems perform, where virtual and real videobronchoscopes simultaneously navigate through distinct manifestations of the same physical chest space [14,38]. For our problem, image guidance involves synchronizing the two spaces during both videobronchoscopic navigation and EBUS localization. In this way, the location of the real device can be confirmed at all times.

During the live procedure, the system used a multimodal virtual EBUS bronchoscope, which extends the concept of the CT-based virtual bronchoscope. This tool’s multimodal display mimics both components of the real EBUS bronchoscope, as shown in Figure 3. The CT-based virtual videobronchoscope simulated the views supplied by the real device’s videobronchoscope camera, as in existing image-guided bronchoscopy systems. In addition, a CT-based virtual EBUS probe simulated 2D fan-shaped EBUS views mimicking the scan plane of the real EBUS probe.

**Figure 4:**
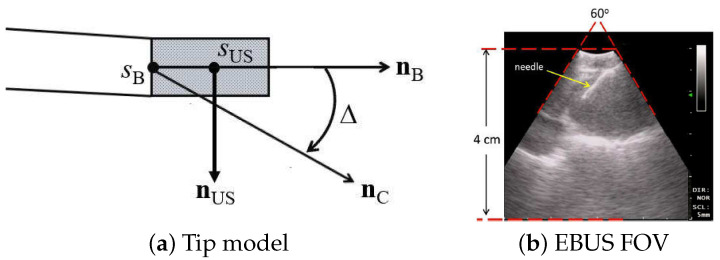
EBUS probe model: (**a**) device tip model; (**b**) 2D EBUS probe view; red lines denote the 60${}^{\circ }$ fan-shaped view. Standard EBUS display settings were used throughout (gain = 19; contrast = 6).

We designed the virtual device’s videobronchoscope camera and EBUS probe to comply with the known geometry and specifications of the Olympus BF-UC180F. Both components are at 3D orientations identical to the configuration of the real EBUS bronchoscope, and both components have identical FOVs of the real device. We give the specific design parameters below, per Figure 4:

- Bronchoscope tip starts at point $s_{B}$ with axis $n_{B}$.
- Videobronchoscope camera axis = $n_{C}$, offset by an angle $\Delta =20^{\circ }$ from $n_{B}$.
- EBUS probe axis $n_{US}$⊥$n_{B}$ at a distance 6 mm from the tip start $s_{B}$; i.e., $\left|\right|s_{B}-s_{US}\left|\right|=6$ mm, where $s_{US}$ is the origin of $n_{US}$.
- 2D EBUS fan-shaped scan plane sweep = 60${}^{\circ }$ with range = 4 cm.

With reference to Figure 1, the magenta arrow represents the virtual bronchoscope’s camera axis $n_{C}$, and the blue arrow represents the virtual EBUS probe axis $n_{US}$. In addition, per Figure 3, in virtual EBUS view $I_{\mathrm{CT}}$, the blue lines delineate the virtual EBUS’s fan-shaped field FOV in $I_{\mathrm{CT}}$, whereas the magenta line denotes the camera axis $n_{C}$. In this way, the virtual EBUS bronchoscope gives a mechanism for relating the two live data streams to the CT-based virtual space. In particular, it provides the necessary linkage to enable image-guided EBUS bronchoscopy.

For a given lymph node, the physician follows the system display as follows. First, the physician *navigates* the “real” EBUS bronchoscope close to the node along the preplanned airway guidance route by following the standard virtual bronchoscope. This step draws on an established approach for registering live bronchoscopic video to CT-based VB views (e.g, Merritt et al. [14])—this synchronizes the positions of both the virtual and real devices. Next, the physician follows the virtual EBUS display to localize the lymph node via EBUS. As discussed in Section 2.2, this second step critically hinges on our proposed CT-EBUS registration method, which synchronizes the positions of the virtual and real EBUS probes. Section 3 later highlights the complete system during image-guided bronchoscopy, while Zang et al. gives more system detail [26].

### 2.2. Virtual-to-Real EBUS Registration

Proper localization of a lymph node depends on our proposed method for registering the virtual and real EBUS probes. To this point, registration involves aligning the virtual EBUS view derived from the preoperative CT scan and the live “real” intraoperative EBUS probe view.

For our EBUS bronchoscopy problem, we performed virtual-to-real EBUS registration by solving an optimization problem. Let $I_{\mathrm{US}}$ denote the target EBUS view in 3D real space and $I_{\mathrm{CT}}^{p}$ denote a CT-based virtual EBUS view at pose *p* within the 3D virtual space, where

(1) $$p=\left\{a,b,c,t_{x},t_{y},t_{z}\right\}\;,$$

(a,b,c) denote the Euler angles, and $\left(t_{x},t_{y},t_{z}\right)$ denote the 3D position. Per Figure 4a, *p* specifies the location $s_{US}$ and probe axis direction $n_{US}$ of the virtual EBUS probe. Upon reaching a lymph node’s general vicinity via standard videobronchoscopy guidance as specified by known pose $p_{i}$ along the preplanned airway guidance route (e.g., using [14]), the physician next pushes the EBUS probe against the airway wall to give view $I_{\mathrm{US}}$. The pose of $I_{\mathrm{US}}$ is technically unknown, since the physician manually performs this scan at their discretion. However, assuming that the physician follows the cues supplied by the guidance system during the guided procedure, we can surmise that the pose of $I_{\mathrm{US}}$ is close to the known pose $p_{i}$ of view $I_{\mathrm{CT}}^{p_{i}}$. Hence, pose *p* in (1) is initialized to known pose $p_{i}$ in virtual space. This is the starting point of our optimal registration problem.

To formulate the optimization, we employed a cost function C that combines raw image intensity information and known ROI segmentation knowledge. In particular,

(2) $$C\left(I_{\mathrm{CT}}^{p},I_{\mathrm{US}}\right)=C_{N}\left(I_{\mathrm{CT}}^{p},I_{\mathrm{US}}\right)-C_{D}\left(I_{\mathrm{CT}}^{p},I_{\mathrm{US}}\right)\;,$$

where $C_{N}$ is the NMI metric, focusing on intensity information, and $C_{D}$ is the Dice index, focusing on ROI shape knowledge. The NMI metric, adapted from Helferty et al. [39], is given by

(3) $$C_{N}\left(I_{\mathrm{CT}}^{p},I_{\mathrm{US}}\right)=1-\frac{h\left(I_{\mathrm{CT}}^{p}\right)+h\left(I_{\mathrm{US}}\right)}{h(I_{\mathrm{CT}}^{p},I_{\mathrm{US}})}\;,$$

whereas the Dice index, a commonly used measure of ROI overlap between two images [40,41], is given by

(4) $$C_{D}\left(I_{\mathrm{CT}}^{p},I_{\mathrm{US}}\right)=\frac{2|R_{\mathrm{CT}}^{p}\cap R_{\mathrm{US}}|}{|R_{\mathrm{CT}}^{p}\left|+\right|R_{\mathrm{US}}|}\;.$$

In (3), $h(I_{\mathrm{CT}}^{p})$ and $h(I_{\mathrm{US}})$ are marginal entropies of images $I_{\mathrm{CT}}^{p}$ and $I_{\mathrm{US}}$, whereas $h(I_{\mathrm{CT}}^{p},I_{\mathrm{US}})$ is a joint entropy:

(5) $$\begin{array}{cc} h(I_{\mathrm{CT}}^{p}) & =-\sum_{k=0}^{M-1}\sum_{l=0}^{M-1}P_{\mathrm{CT},\mathrm{US}}\left(k,l\right)\log P_{\mathrm{CT}}\left(k\right)\\ h(I_{\mathrm{US}}) & =-\sum_{k=0}^{M-1}\sum_{l=0}^{M-1}P_{\mathrm{CT},\mathrm{US}}\left(k,l\right)\log P_{\mathrm{US}}\left(l\right)\\ h(I_{\mathrm{CT}}^{p},I_{\mathrm{US}}) & =-\sum_{k=0}^{M-1}\sum_{l=0}^{M-1}P_{\mathrm{CT},\mathrm{US}}\left(k,l\right)\log P_{\mathrm{CT},\mathrm{US}}\left(k,l\right) \end{array}$$

where $P_{\mathrm{CT}}\left(\cdot \right)$ and $P_{\mathrm{US}}\left(\cdot \right)$ are the respective marginal probability density functions of the pixel intensity values in images $I_{\mathrm{CT}}^{p}$ and $I_{\mathrm{US}}$, $P_{\mathrm{CT},\mathrm{US}}\left(\cdot ,\cdot \right)$ is the joint density function between corresponding pixels in the two images, and M=256 is the number of gray levels that an image pixel can assume. In (4), $R_{\mathrm{CT}}^{p}$ represents the target lymph node’s region of interest (ROI) as it appears in virtual EBUS view $I_{\mathrm{CT}}^{p}$; i.e.,

(6) $$\begin{array}{cc} R_{\mathrm{CT}}^{p}\left(x,y\right) & =\left\{\begin{array}{cc} 1, & \mathrm{if}\;I_{\mathrm{CT}}^{p}\left(x,y\right)\in \mathrm{target}\;\mathrm{node’s}\;\mathrm{ROI}\;\mathrm{in}\;\mathrm{CT}\\ 0, & \mathrm{otherwise}\;\;\; \end{array}\right.\;, \end{array}$$

where the ROI was defined during planning. Similarly, $R_{\mathrm{US}}$ equals the segmented ROI appearing in EBUS frame $I_{\mathrm{US}}$, where we used the previously validated automatic method of Zang et al. for this operation [42]. Thus, the collection of CT scan voxels constituting $R_{\mathrm{CT}}^{p}$, as given by (6), corresponds to the known ROI knowledge that will be correlated with $R_{\mathrm{US}}$ during optimization. Cost C of (2) is in the range $-2\leq C(I_{\mathrm{CT}}^{p},I_{\mathrm{US}})\leq 1,$ since $C_{N}\left(I_{\mathrm{CT}}^{p},I_{\mathrm{US}}\right)$ ranges from −1 (strongest NMI correlation) to 1 (no correlation) and $C_{D}\left(I_{\mathrm{CT}}^{p},I_{\mathrm{US}}\right)$ ranges from 0 (no ROI overlap) to 1 (total ROI overlap).

Given ((2)–(6)), our optimization problem was formulated as

(7) $$p_{o}=\arg\left\{\min_{p\in N_{p_{i}}}C\left(I_{\mathrm{CT}}^{p},I_{\mathrm{US}}\right)\right\}\;,$$

where $p_{i}$ is the initial pose, $N_{p_{i}}$ is a search neighborhood around $p_{i}$, and $p_{o}$ is the optimal pose minimizing cost C(·,·). To solve (7), we adapted the simplex optimization algorithm to our EBUS registration problem to iteratively search for pose $p_{o}$ giving minimum cost C [43]. To this search, we added a constraint that took into account that the physician must push the EBUS probe against the airway wall to acquire an EBUS view. This implies that $p_{i}$ and poses $p\in N_{p_{i}}$ must be situated on the airway wall surface. Therefore, we interleaved a surface-voxel search with the simplex algorithm. We now summarize the complete Algorithm 1.
**Algorithm 1:** Multimodal CT-EBUS Registration Algorithm.1.Initialize optimization (7) with captured EBUS frame $I_{\mathrm{US}}$ and VB view $I_{\mathrm{CT}}^{p_{i}}$ at pose $p_{i}$.2.Segment ROI $R_{\mathrm{US}}$ in $I_{\mathrm{US}}$ using the method of Zang et al. [42].3.Derive a modified pose $p_{i}$ and a simplex $N_{p_{i}}$ that constrains the possible candidate neighboring poses to be situated on the airway wall, as depicted in the chest CT scan.4.Using cost function C(·,·) defined by ((2)–(6)) and simplex $N_{p_{i}}$, run the simplex algorithm on (7) to update pose $p_{o}$ until it does not change for *T* iterations.5.Repeat steps 3–4 using $p_{i}=p_{o}$ until $p_{o}$ no longer changes.6.Output $p_{o}$ and virtual EBUS view $I_{\mathrm{CT}}^{p_{o}}$.

At the conclusion of the registration algorithm, we now know the precise location in 3D chest space of the lymph node depicted in the live intraoperative EBUS probe view $I_{\mathrm{US}}$, as specified by $p_{o}$. We elaborate on the algorithm’s steps below.

**Figure 5:**
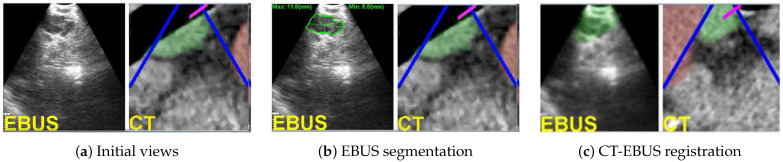
CT-EBUS registration for a station-10 lymph node (case 21405-139). (**a**) Initial raw EBUS view $I_{\mathrm{US}}$ (left) and corresponding CT-based virtual EBUS view $I_{\mathrm{CT}}^{p_{i}}$ (right). (**b**) Result after segmenting the nodal ROI $R_{\mathrm{US}}$ in EBUS view $I_{\mathrm{US}}$; the green outline signifies the ROI contour [42]. (**c**) Registered pair ($I_{\mathrm{US}}$, $I_{\mathrm{CT}}^{p_{o}}$) after final registration; $I_{\mathrm{US}}$ depicts the fused registered CT-based ROI. In all virtual EBUS views, green regions denote nodal ROIs $R_{\mathrm{CT}}$ predefined during planning, whereas red regions represent blood vessels.

Regarding steps 1–2, Figure 5a,b depicts an example of initial real and virtual EBUS views $\left\{I_{\mathrm{US}},I_{\mathrm{CT}}^{p_{i}}\right\}$ for a station-10 lymph node. For the real space, $I_{\mathrm{US}}$ is initialized as an EBUS frame captured after completing bronchoscopic navigation. For the virtual space, $p_{i}$ is the known final pose reached during navigation, whereas $R_{\mathrm{CT}}^{p_{i}}$ equals the predefined nodal ROI as it appears in $I_{\mathrm{CT}}^{p_{i}}$ (green regions in right-side views of Figure 5a–c).

Step 3 next generates a simplex $N_{p_{i}}$ of suitable candidate search poses, where $N_{p_{i}}$ is defined by seven vertices situated on the airway wall within CT-based virtual space. Each simplex vertex represents a pose on the airway wall neighboring $p_{i}$. To begin, we first derived a modified pose $p_{i}$ as the first voxel along the virtual EBUS device’s viewing axis $n_{US}$ whose value was >−600 HU (HU = Hounsfield units). In chest CT images, it is well known that air appears as dark voxels with HU value ≈−1000 and the brighter surrounding airway walls have HU values in the range [50, 200]. Hence, we picked the conservative threshold −600 to identify potential airway wall voxels [35]. Next, to derive simplex $N_{p_{i}}$, we first computed six candidate vertices $p_{v}$ defining the simplex by changing the six parameters of $p_{i}$ separately with increments Δa, Δb, Δc, $\Delta t_{x}$, $\Delta t_{y}$, and $\Delta t_{z}$, per (1) [44]. We then adjusted the vertices $p_{v}$ to corresponding surface points on the airway wall.

**Figure 6:**
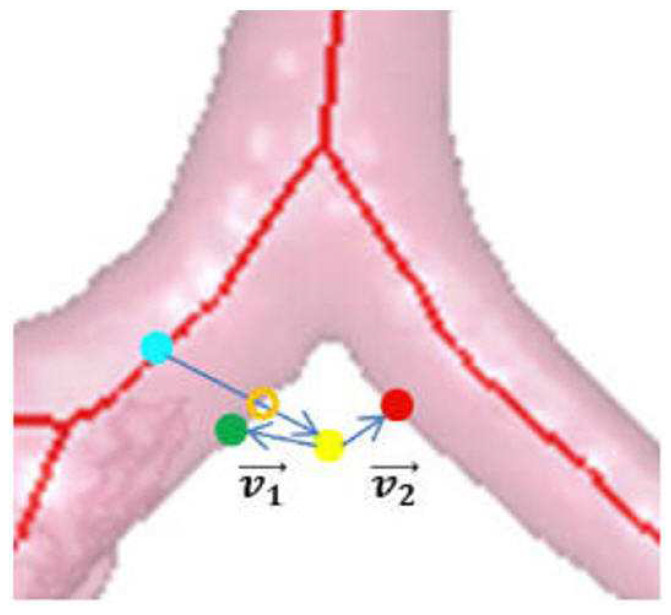
Locating airway wall point $p_{s}$. Green dot denotes the current surface point $p_{i}$, yellow dot denotes the candidate position $p_{v}$, red dot is the closest k-d tree point $p_{s}$ to $p_{v}$, cyan dot is the closest airway centerline point $p_{l}$ to $p_{v}$, and orange hollow dot is the correct surface voxel $p_{s}^{\prime }$.

To find the surface points defining the simplex, we used the algorithm of Gibbs et al. [35]. The search drew upon the airway-tree surface-voxel structure constituting the previously derived airway-tree endoluminal surfaces. To begin, we constructed a k-d tree structure for these voxels to facilitate a quick search. Subsequently, for each vertex $p_{v}$ of the initial simplex, we searched the k-d tree for the point $p_{s}$ closest to $p_{v}$. Since the marching cubes algorithm was used to compute the airway-tree endoluminal surfaces, it had a thickness of 1 layer [45]. However, larger airways have walls of thickness >1 voxel; this introduces uncertainty into the surface voxel locations [35]. Thus, to account for thicker airway walls, we also considered neighboring voxels surrounding surface point $p_{s}$ as valid candidate vertices. In particular, if $p_{v}$ has HU value above the surface threshold and $\left|\right|p_{v}-p_{s}\left|\right|<1$ mm, then we accepted $p_{v}$ as an adjusted vertex. Note, however, that the k-d tree was based only on surface voxel coordinates, without regard for airway location. Hence, the closest $p_{s}$ to a given $p_{v}$ could be on the wrong airway tree branch, as Gibbs et al. noted [35]. To ensure that $p_{s}$ is on the same airway branch as $p_{v}$, we performed the following, per Figure 6:

1. Compute dot product ${\vec{v}}_{1}\cdot {\vec{v}}_{2}$, where ${\vec{v}}_{1}=p_{v}-p_{i}$ and ${\vec{v}}_{2}=p_{v}-p_{s}$.
2. If ${\vec{v}}_{1}\cdot {\vec{v}}_{2}>0$, $p_{s}$ is part of the correct airway and is kept as the updated vertex.
3. Otherwise, $p_{s}$ is from the wrong branch. Find the closest airway centerline point $p_{l}$ to $p_{i}$ and search for a new surface voxel candidate $p_{s}^{\prime }$ based on the HU threshold along the direction from $p_{l}$ to $p_{v}$.

The six adjusted vertices $p_{v}$ along with $p_{i}$ delineate the initial simplex $N_{p_{i}}$ for optimization step 4.

Step 4 now applies the iterative simplex algorithm to collapse the simplex around an optimal pose $p_{o}$ [43,44]. During each iteration, cost C was evaluated for all vertices. Depending on these results, various geometric operations were performed that either expand, contract, or reflect the simplex vertices toward a minimum cost voxel solution $p_{o}$. The algorithm continued until $p_{o}$ stayed unchanged for *T* iterations. Steps 3–4 of the top-level multimodal CT-EBUS registration algorithm were then repeated until the voxel corresponding to $p_{o}$ no longer changed.

**Figure 7:**
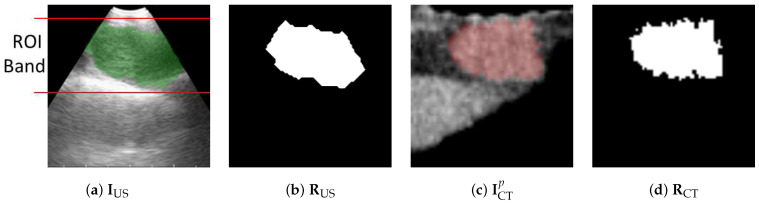
Limiting region of calculations for (5) in cost $C_{N}$. (**a**) Trapezoidal EBUS region delineated by the lines encompassing the EBUS ROI in $I_{\mathrm{US}}$ with (**b**) showing segmented ROI $R_{\mathrm{US}}$. (**c**,**d**) Corresponding virtual EBUS view $I_{\mathrm{CT}}^{p}$ and predefined ROI $R_{\mathrm{CT}}$.

The marginal densities (5) used by cost function $C_{N}$ in ((2)–(3)) were estimated by normalized image histograms of images $I_{\mathrm{CT}}^{p}$ and $I_{\mathrm{US}}$, while the joint density was given by the normalized joint histogram between the two images. These calculations, however, did not use an entire image. Since EBUS images are noisy and filled with bland low-information regions, major portions of $I_{\mathrm{CT}}^{p}$ and $I_{\mathrm{US}}$ provided misleading/useless information. Instead, we first defined the smallest trapezoid that bounds the segmented ROI in the given image $I_{\mathrm{US}}$ and stays within the EBUS’s fan-shaped scan region. This region contains the most useful findings. Next, for both $I_{\mathrm{CT}}^{p}$ and $I_{\mathrm{US}}$, we only used pixels within the bounding trapezoid to calculate the required histograms; see Figure 7.

For the station 10 node example of Figure 5, the final registration (Figure 5c) required 57 iterations of steps 3–5. The result clearly confirms that the physician has settled at an effective site. Figure 3 gives another registration example for a station 4R node (case 21405-116).

### 2.3. Implementation

We ran all experiments on the guidance computer. For our tests, this computer was a Dell Precision T5500 64-bit Windows-based PC (dual 2.8 GHz 6-core CPUs, 24 GB RAM) powered by an Nvidia Quadro 4000 2GB PCIe graphics engine and a Matrox Vio IA/OA frame grabber. All software was implemented in C++. Many data-intensive operations were parallelized using Nvidia’s compute-unified device-architecture (CUDA) tools and OpenMP. A 24-inch Dell monitor served as the guidance system’s display. A standard Olympus Evis Exera III bronchoscopy suite was used for all tests (CV-190 video system, CLV-190 light source, EU-ME1 ultrasound unit, and display monitor). To interface the EBUS bronchoscope to the guidance computer, we connected a BNC-to-BNC cable from the bronchoscope display monitor’s PnP (picture-in-picture) video output to the guidance computer’s Matrox video input. This gave access to the live video streams from the EBUS bronchoscope (both the bronchoscopic video and EBUS).

## 3. Results

Section 3.1 tests the efficacy of our virtual-to-real EBUS registration method, whereas Section 3.2 presents example results using our method within a complete image-guided EBUS bronchoscopy system.

### 3.1. CT-EBUS Registration Study

We developed and tested our method using data collected retrospectively from 10 lung-cancer patients. These data were collected at our University’s lung cancer management clinic through two IRB-approved study protocols under informed consent. Chest CT scans were generated by Siemens CT scanners. Each scan was made up of 512 × 512 axial-plane sections (number of sections per scan: 570 to 720), with section thickness = 0.75 mm, section spacing Δz = 0.5 mm, and axial-plane resolution Δx=Δy ranging from 0.60 mm to 0.81 mm. Seven studies drew upon a standard definition EBUS bronchoscope video feed giving 300 × 300 EBUS views, and three studies drew upon a high-definition video feed giving 816 × 848 EBUS views. Over the 10 patients, 28 ROIs were predefined, with a typical long axis length >15 mm: 27 lymph nodes and 1 azygos vein. The lymph nodes were distributed over the nodal stations as follows: station 4, 10 nodes; station 7, 9 nodes; station 10, 5 nodes; and station 11, 3 nodes.

To define the ground truth for each ROI, we first picked a 2D EBUS frame $I_{\mathrm{US}}$ that depicts the ROI. Next, we performed 2D EBUS segmentation using Zang’s method to extract the ROI in $I_{\mathrm{US}}$ [42]. Finally, we established the ground-truth virtual EBUS view $I_{\mathrm{CT}}^{p_{G}}$ best matching $I_{\mathrm{US}}$. To achieve this, we started at the final pose $p_{i}$ of the ROI’s precomputed optimal airway route in CT-based virtual space [25]. We then manually moved the virtual EBUS bronchoscope around this pose to locate a ground-truth pose $p_{G}$ such that view $I_{\mathrm{CT}}^{p_{G}}$ most closely mimics the anatomical appearance shown in $I_{\mathrm{US}}$.

To perform the test for each ROI, we began by running the registration method at initial pose $p_{i}$ in virtual space. Upon convergence, the method returned a virtual EBUS view $I_{\mathrm{CT}}^{p}$ at an optimal pose $p=p_{o}$. Let

$$p_{G}=\left[t_{x}^{G},\;t_{y}^{G},\;t_{z}^{G}\right]\;,\;p_{o}=\left[t_{x}^{o},\;t_{y}^{o},\;t_{z}^{o}\right]$$

represent the 3D positions and

$$d_{G}\left(a^{G},\;b^{G},\;c^{G}\right)\;,\;d_{o}\left(a^{o},\;b^{o},\;c^{o}\right)$$

denote the direction vectors for $p_{G}$ and $p_{o}$, respectively. To quantify the registration performance, we compared $p_{o}$ and $p_{G}$ using three metrics, as suggested in [14]:

1. Position difference $e_{p}$, which measures the Euclidean distance between $p_{o}$ and $p_{G}$:
  (8) $$e_{p}=\left|\right|p_{o}-p_{G}\left|\right|$$
2. Direction error $e_{d}$, which gives the angle between $d_{o}$ and $d_{G}$:
  (9) $$e_{d}=\cos^{-1}\left(d_{o}\cdot d_{G}\right)$$
3. Needle difference $e_{N}$, which indicates the distance between two extended needle tips at $p_{o}$ and $p_{G}$:
  (10) $$e_{N}=\left|\right|\left(p_{o}+l_{N}d_{o}\right)-\left(p_{G}+l_{N}d_{G}\right)\left|\right|$$

Regarding these metrics, $e_{p}$ indicates the positional error of the bronchoscope on the airway surface, $e_{d}$ quantifies the orientation error, and $e_{N}$ measures the potential biopsy error.

**Figure 8:**
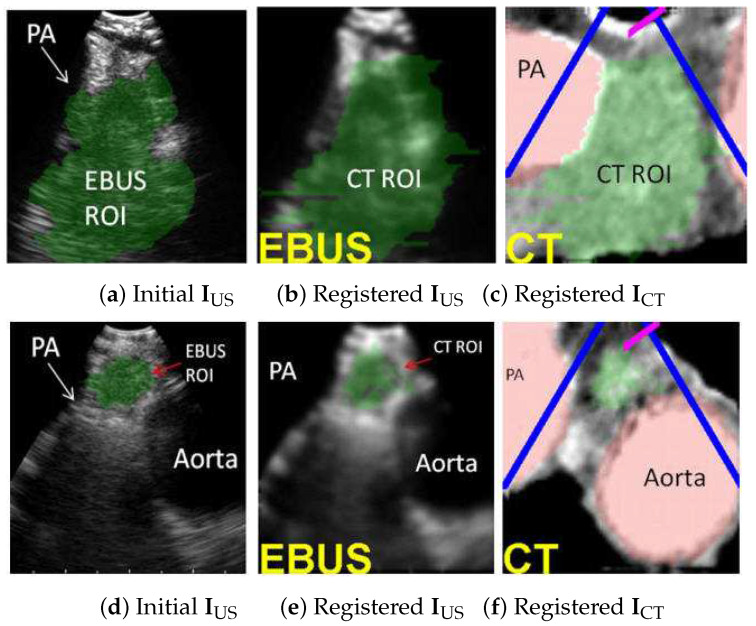
CT-EBUS registration examples. (**a**–**c**) Station 4L node for case 20349-3-84. (**d**–**f**) Station 4L node for case 21405-108. Parts (**a**,**d**) show the automatically segmented ROI in EBUS frame $I_{\mathrm{US}}$ using [42]. Parts (**b**,**e**) show the registered CT-based predefined ROI superimposed on $I_{\mathrm{US}}$. Parts (**c**,**f**) depict the CT-based virtual EBUS view $I_{\mathrm{CT}}^{p_{o}}$ after registration. In all views, the green region corresponds to the lymph node, whereas the red regions represent major vessels (PA = pulmonary artery).

For the 28-ROI test set, the registration method correctly localized 28/28 ROIs (100%), with average registration time = 3.4 s, which represents the time to complete the iterative simplex optimization algorithm for (7) after segmenting the ROI in the captured EBUS frame $R_{\mathrm{US}}$. (We chose T=15 for this test.) Table 1 gives the aggregate performance for the error metrics. Lastly, Figure 8 illustrates two registration examples. The results firmly assert the effectiveness of the method.

We next performed a parameter sensitivity test for the registration method. For the test, we first randomly picked one ROI from each human case used in the 28-ROI study. We then performed tests on these 10 ROIs, whereby one search parameter was varied over a given range:

1. Positional parameters $\Delta t_{x}$, $\Delta t_{y}$, and $\Delta t_{z}$, range [−10 mm, 10 mm], step size = 2.5 mm.
2. Angle parameters Δa, Δb, and $\Delta_{c}$, range[−100${}^{\circ }$, 100${}^{\circ }$], step size = 25${}^{\circ }$.
3. Iteration parameter *T* from 5 to 25, step size 5.

(The angle range accounts for the physician’s limitations in twisting the bronchoscope [46]). For each test, one parameter was changed, whereas all others were held at the default values. Each run always started at the ground truth pose $p_{i}$ that terminates an ROI’s derived optimal path.

Table 2, Table 3 and Table 4 summarize the results for three parameters; results for the other parameters are similar. All results are given over the 10-ROI test set. As a disclaimer, we point out that technician judgment was required in interactively deriving the “best matching” virtual EBUS bronchoscope views serving as the ground truth. As a result of the inherent degradations in EBUS views (broken edges, speckle, low detail content), it was difficult to locate the single pose for which the virtual EBUS view “best” matches the real EBUS frame. This adds bias to our ground truth. In addition, ambiguity can exist when registering a 2D EBUS image to a 3D CT subvolume because the lymph nodes often have approximately spherical/elliptical 3D shapes. Thus, results can vary over different initial conditions.

Overall, the initial condition step sizes, which affect the size of the initial simplex, had a minimal impact on the performance. The bronchoscope position and needle errors, $e_{p}$ and $e_{N}$, were generally always under 5 mm and 9 mm, respectively, which are smaller than the typical clinically considered lymph node (long axis > 10 mm). The angle error $e_{d}$ also fluctuated under 25${}^{\circ }$. Larger errors are attributable to the aforementioned ambiguity. Increasing iteration number *T* beyond five iterations does result in performance improvement. This gain was less significant, however, from 15 to 25 iterations, especially considering that the mean execution time increased from 3.4 to 8.0 s. Hence, for the previous 28-ROI study and all later human studies, we chose T=15 as the default iteration number.

Regarding the 28-ROI test, the precise mean registration time = 3.4 s ± 1.6 s, with a range = [1.9 s, 10.0 s]. Notably, the registration time does depend somewhat on the size of the node. We note that the two largest nodes considered required the longest registration times: (1) 10.0 s for a station 7 node with all axes ≥2.4 cm (long axis = 3.1 cm); and (2) 7.9 s for a station 4R node with all axes ≥ 1.6 cm (long axis = 2.7 cm). Excluding these two outliers, the mean registration time for the other 26 test nodes = 3.0 s ± 0.5 s (range, [1.9 s, 4.1 s]. As the discussion later highlights, these times are acceptable for real usage. Overall, these results support the method’s robustness.

### 3.2. Image-Guided EBUS Bronchoscopy System

**Figure 9:**
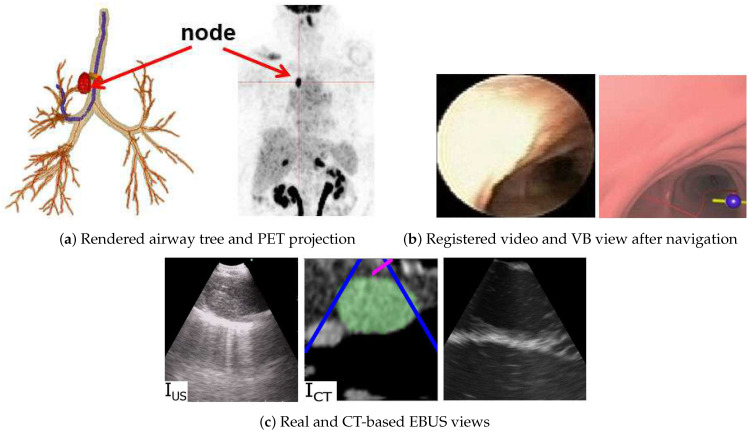
Image-guided EBUS bronchoscopy for a station 4R lymph node for patient 21405-116. (**a**) 3D airway tree rendering and whole-body PET projection image indicating the target lymph node (red). (**b**) Registered real video and VB view after navigation. (**c**) Registered real EBUS view $I_{\mathrm{US}}$, virtual EBUS view $I_{\mathrm{CT}}$ (green region = node), and a CT-based simulated EBUS view, respectively, at final site.

The registration method has been integrated into a complete image-guided EBUS bronchoscopy system, as described more fully in [26]. Figure 9 illustrates a retrospective example of system guidance for a 68-year-old female lung cancer patient presenting a station 4R lymph node. Both a high-resolution chest CT and a whole-body PET/CT study were available. The chest CT, produced by a Siemens SOMATOM Definition Flash, has specifications Δx=Δy= 0.77 mm, Δz= 0.5 mm, and volume dimensions = 512 × 512 × 570, while the PET/CT study, generated by a Philips TrueFlight integrated scanner, provided a PET scan with Δx=Δy= 4 mm, Δz= 3 mm, and volume dimensions = 144 × 144 × 284. The example depicts both the videobronchoscopic navigation and EBUS localization guidance phases. Figure 9c clearly shows successful registration during the EBUS localization of the node. Notably, Figure 9c also depicts a CT-based simulated EBUS view, generated by the method in [47]. While CT-EBUS registration is performed using the CT-based EBUS view, the supplemental EBUS simulation, which strongly resembles the real EBUS view, adds confidence in the attained position of the real EBUS probe.

**Figure 10:**
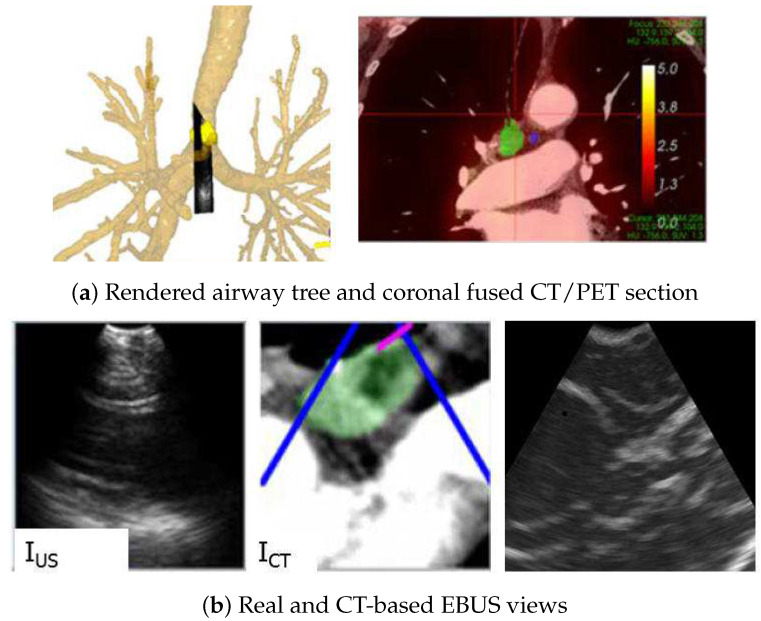
Image-guided EBUS bronchoscopy for a station 4R lymph node for patient 20349-3-87. (**a**) 3D airway tree rendering and coronal fused CT/PET section indicating the target lymph node (color scale bar indicate PET SUV value). (**b**) Registered real EBUS view $I_{\mathrm{US}}$, virtual EBUS view $I_{\mathrm{CT}}$ (green region = node), and a CT-based simulated EBUS view, respectively, at final site.

Figure 10 gives a second example from a live prospective guided procedure for a 77-year-old female lung cancer patient presenting a station 4R node. As with the previous example, both a high-resolution chest CT and a whole-body PET/CT study were available. The chest CT, produced by a Siemens SOMATOM Definition Flash, has specifications Δx=Δy= 0.57 mm, Δz= 0.5 mm, and volume dimensions = 512 × 512 × 663, while the PET/CT study, again generated by a Philips TrueFlight integrated scanner, provided a PET scan with Δx=Δy= 4 mm, Δz= 3 mm, and volume dimensions = 144 × 144 × 284. The composite display view during EBUS localization indicates that the EBUS bronchoscope has reached the target node. In addition, the virtual and real EBUS views align well, with the simulated view corroborating the expected appearance of the real view.

## 4. Discussion

Lung cancer persists as the most common type of cancer death, with a mortality rate >85% [48]. The staging of the chest lymph nodes via EBUS bronchoscopy is one of the major steps in managing a lung cancer patient. While physicians can readily navigate the device close to the correct vicinity of a lymph node—i.e., get into the right “room” (airway)—their subsequent attempt to then correctly localize the node with EBUS and then perform an adequate biopsy—i.e., hit the right wall in the room (airway wall)—is well-known to be problematic.

We proposed a near real-time CT-EBUS registration method that facilitates an accurate EBUS-based examination of a lymph node during an EBUS bronchoscopy. In particular, after the physician navigates the bronchoscope near the lymph node’s vicinity, the method enables accurate image-guided EBUS probe placement; i.e., the physician can immediately localize the lymph node without the uncertainty encountered in standard EBUS usage. Laboratory results using data from lung cancer patients show the registration method’s robust performance.

The method has also been integrated into a complete image-guided EBUS bronchoscopy system designed for chest nodal staging, as demonstrated with system examples. A companion paper by Zang et al. gives more detail related to the system’s guidance and display capabilities [26]. As discussed fully in the companion paper, the system’s functionality and feasibility has been validated in both retrospective and prospective human studies at our University Hospital. For the 13-patient prospective study run within our hospital’s standard clinical work flow, 100% of preselected target lymph nodes were successfully localized using the system, a strong indicator of our registration method’s efficacy. The mean time for performing image-guided EBUS for a particular node was 87.4 s, with a total mean procedure time of 6 min 43 s (4.61 nodes per patient). This time includes all time for device navigation, EBUS localization, EBUS segmentation, and final registration, and it excludes biopsy time. In addition, the system appeared to be safe and feasible in the live clinical setting, with no adverse events reported. Complete detail of this study appears in [26].

While these results point to the potential practicality of the method for live clinical practice, a larger multi-center study is needed to more fully ascertain our methodology’s utility for enabling more efficacious EBUS-based nodal staging. One notable limitation is the need to segment ROIs live in the captured real EBUS frame. Some ROIs required multiple segmentation attempts. However, the prospective study gave an acceptable 18.1 s mean time to segment an ROI in a real EBUS frame (part of the 87.4 s mean procedure time per node). Finally, the system requires integration into an approved system meeting federal FDA quality standards.

As an additional point, for all lymph nodes considered in our studies, the physician specified the nodal station labels while selecting target lymph nodes on CT during procedure planning. Hence, during the later image-guided EBUS bronchoscopy procedure, when a node scanned by the “real” EBUS is registered to the target CT-based node, this not only confirms the 3D physical location of the node, but it also implicitly confirms the real node’s station label. While not a part of this paper, we had devised a methodology previously for automatically defining the nodal stations and assigning station labels to lymph nodes identified in CT; e.g., [49,50].

On another note, we also easily adapted the registration method to the newer Olympus BF-UC190F EBUS bronchoscope by a simple adjustment of scope tip specifications [51]. By making a similar adjustment, other related devices, such as a recently proposed thinner EBUS bronchoscope capable of going deeper into the airway tree [52], could also be guided using our methodology. Finally, our methodology could help to drive robotics-based bronchoscopy systems, which currently offer assistance for the bronchoscope only [10].

## Figures and Tables

**Table 1:** Registration performance over the 28-ROI test set.

Metric	Mean ± Std. Dev.	[Low, High]
$e_{p}$ (mm)	2.2 mm ± 2.3 mm	[0.2 mm, 11.8 mm]
$e_{N}$ (mm)	4.3 mm ± 3.0 mm	[1.1 mm, 11.7 mm]
$e_{d}$ (${}^{\circ }$)	11.8${}^{\circ }$ ± 8.8${}^{\circ }$	[0.4${}^{\circ }$, 41.3${}^{\circ }$]

**Table 2:** Registration sensitivity to variation in $\Delta t_{x}$.

$\Delta t_{x}$ (mm)	$e_{p}$ (mm)		$e_{N}$ (mm)		$e_{d}$ (${}^{\circ }$)	
−10.0	3.7 ± 3.4	[1.1, 11.7]	7.3 ± 5.1	[2.1, 14.6]	18.1 ± 11.1	[4.6, 38.3]
−7.5	4.3 ± 3.3	[1.8, 12.2]	8.4 ± 5.3	[2.5, 18.7]	21.2 ± 15.2	[6, 55.9]
−5.0	4.5 ± 3.5	[1.3, 12.2]	6.4 ± 4	[1.5, 11.4]	18.9 ± 12.7	[5.9, 45.8]
−2.5	4.6 ± 3.1	[1, 10.2]	7.8 ± 4	[2.1, 14.4]	22.5 ± 14.5	[6.2, 46.5]
0.0	3.7 ± 3.2	[1.4, 10.5]	7.8 ± 3.9	[2.7, 14.4]	22.8 ± 12.9	[8.8, 46.2]
2.5	4 ± 2.8	[0.9, 9.4]	8.5 ± 6.7	[1.4, 20.9]	25.9 ± 19.4	[5.7, 61.5]
5.0	3.8 ± 4.5	[0.6, 14.5]	6.4 ± 4.8	[2, 14.9]	19.3 ± 12.1	[5.9, 41.8]
7.5	2.8 ± 3.8	[0.3, 11.8]	4.8 ± 4.5	[1.1, 11.7]	10.8 ± 8.8	[0.4, 22.9]
10.0	3.8 ± 3.9	[1.1, 12]	5.7 ± 4.1	[1.6, 11.7]	16 ± 9.8	[5.5, 30.3]

**Table 3:** Registration sensitivity to variation in Δa.

Δa ${(}^{\circ })$	$e_{p}$ (mm)		$e_{N}$ (mm)		$e_{d}$ (${}^{\circ }$)	
−100.0	6.6 ± 4.2	[2.2, 13.4]	9.0 ± 4.1	[5.4, 14.5]	25.0 ± 12.6	[7.1, 41.2]
−75.0	4.8 ± 4.0	[1.2, 11.5]	8.6 ± 4.8	[3.3, 16.2]	24.2 ± 11.5	[8.0, 40.4]
−50.0	5.0 ± 4.6	[1.3, 14.9]	6.7 ± 3.2	[2.8, 12.3]	17.3 ± 8.8	[5.3, 28.2]
−25.0	3.6 ± 3.6	[0.6, 11.2]	8.2 ± 6.2	[1.3, 20.1]	23.3 ± 13.6	[5.2, 50.8]
0.0	4.4 ± 3.6	[1, 11.8]	8 ± 2.4	[5.5, 12]	26.3 ± 10.3	[15.9, 47.9]
25.0	3.5 ± 2.9	[1, 9.9]	7 ± 3.4	[2.1, 13.4]	20.1 ± 11	[7.8, 36]
50.0	2.8 ± 3.8	[0.3, 11.8]	4.8 ± 4.5	[1.1, 11.7]	10.8 ± 8.8	[0.4, 22.9]
75.0	3.1 ± 3.2	[0.9, 10.4]	6.1 ± 2.8	[3.0, 11.5]	16.5 ± 9.5	[5.6, 29.4]
100.0	3.6 ± 3.2	[1.0, 11.0]	5.0 ± 4.1	[1.4, 10.5]	19.0 ± 11.5	[8.1, 43.7]

**Table 4:** Registration sensitivity to variation in iteration number *T*. Time denotes computation time to complete the optimization.

*T*	$e_{p}$ (mm)		$e_{N}$ (mm)		$e_{d}$ (${}^{\circ }$)		Time (s)
5	3 ± 4.2	[0.9, 13.2]	5.9 ± 5.2	[2, 17.7]	17.3 ± 9.3	[6.4, 36]	1.3
10	2.8 ± 3.8	[0.9, 11.8]	5.3 ± 4.2	[1.3, 11.7]	13.6 ± 7.3	[1.3, 21.9]	2.6
15	2.8 ± 3.8	[0.3, 11.8]	4.8 ± 4.5	[1.1, 11.7]	10.8 ± 8.8	[0.4, 22.9]	3.4
20	3.2 ± 3.9	[0.3, 11.8]	4.4 ± 3.9	[1.1, 11.7]	9.6 ± 7.5	[0.4, 20.5]	5.0
25	2.7 ± 3.8	[0.3, 11.8]	4.2 ± 3.7	[1.1, 11.7]	9.8 ± 7.6	[0.4, 20.5]	8.0

## Data Availability

Not applicable.
